# Vasant Kusumakar Rasa Ameliorates Diabetic Encephalopathy by Reducing Oxidative Stress and Neuroinflammation and Improving Neurotransmitter Levels in Experimental Animals

**DOI:** 10.7759/cureus.75905

**Published:** 2024-12-17

**Authors:** Alok D Singh, Mukesh Chawda, Yogesh A Kulkarni

**Affiliations:** 1 Shobhaben Pratapbhai Patel School of Pharmacy and Technology Management, SVKM’s Narsee Monjee Institute of Management Studies (NMIMS) Deemed to be University, Mumbai, IND; 2 Medical Services, Shree Dhootapapeshwar Limited, Mumbai, IND

**Keywords:** diabetic encephalopathy, high fat diet, neurotransmitters, streptozotocin, vasant kusumakar rasa

## Abstract

Purpose: Diabetic encephalopathy (DE) is one of the complications of diabetes that affects the brain. In the Ayurveda system of medicine, Vasant Kusumakar Rasa (VKR) is cited as a classical herbo-mineral formulation for diabetes. However, the role of VKR in DE is still unclear.

Methods: High-fat diet and streptozotocin (35 mg/kg, *i.p.*) were used to induce type 2 DE in Sprague Dawley rats. VKR at doses 28 mg/kg and 56 mg/kg was given via intragastric route to diabetic rats for 16 weeks. Estimation of plasma glucose, serum insulin, glycohemoglobin, and C-reactive protein (C-RP) was analyzed. Furthermore, the Morris water maze test was performed to assess cognitive behavior. Pro-inflammatory, such as TNF-α, IL-1β, and IL-6, were measured in brain tissue homogenate. Antioxidant enzyme assays were performed to estimate the levels of malondialdehyde, reduced glutathione, superoxide dismutase, and catalase in brain tissue. Histopathology of brain sections was performed using hematoxylin and eosin (H & E) staining. Neurotransmitters (viz., serotonin (5-HT), dopamine (DA), and norepinephrine (NE)) were estimated in the brain by high-performance liquid chromatography (HPLC). The data were analyzed by using ANOVA, followed by Dunnett's multiple comparison test.

Results: VKR treatment, at a dose of 28 and 56 mg/kg, reduced the plasma glucose level significantly (236.7±17.08 and 221.8±17.50, respectively; p<0.001) when compared with diabetic control (461.7±13.03). The treatment also reduced serum insulin and glycated hemoglobin levels and improved the escape latency in VKR-treated animals as compared to diabetic animals. Brain tissue pro-inflammatory marker levels were reduced, and oxidative stress enzymes showed positive marks in diabetic rats treated with VKR. Histopathology of the brain demonstrated a reduction in neuronal damage in the VKR-treated diabetic animals. VKR treatment at doses of 28 and 56 mg/kg also improved the levels of 5-HT (1.78±0.11 and 1.72±0.18, respectively) when compared with diabetic control (0.91±0.08) significantly (p<0.01). DA levels were significantly (p<0.01) increased in VKR-treated animals when compared with diabetic animals. The treatment of VKR for 16 weeks also improved the NE levels significantly when compared with diabetic control animals.

Conclusion: The result of the study indicates that the treatment with VKR for 16 weeks has significant therapeutic potential in the management of type 2 DE.

## Introduction

Diabetic encephalopathy (DE) is a chronic condition associated with long-term hyperglycemia, causing structural and physiological damage to the central nervous system (CNS) [1]. One of the lesser-known but serious complications that can arise as a result of this diabetic condition is DE, a neurological disorder that remains insufficiently acknowledged and understood in the context of diabetes and its multifaceted effects on cognitive function and overall brain health [2].

The clinical hallmark of DE includes altered cognitive function, motor impairment, and dementia [1]. The pathophysiological characteristics of DE are associated with the alteration in glucose homeostasis, increased neuroinflammation, oxidative stress generation, mitochondrial dysfunction, demyelination, blood-brain barrier leakage, and dysregulation of neurotransmitters within the brain region [3-5]. These imbalances diminish neuronal survival and conduction, which contributes to the development of glucose-associated neurotoxicity [6]. Diabetic patients are at higher risk of developing dementia as compared to non-diabetic individuals, resulting in poor quality of life among the diabetic population [7]. The precise mechanism of DE remains poorly comprehended, yet it is primarily linked to prolonged hyperglycemia [8]. Progression of DE can be delayed by the utilization of antioxidants, acetylcholinesterase (AChE), sulfonylurea, C-peptide, and other clinically approved newer antidiabetic agents. Nonetheless, the pharmaceutical agents are associated with a range of adverse effects and do not treat completely [9]. As of now, there is currently no medication that has been specifically developed for the purpose of preventing or managing DE. Ayurveda is an ancient Indian system of medicine that encompasses a combination of herbal, mineral/metal, and animal-derived substances that undergo processing to yield therapeutic benefits.

Vasant Kusumakar Rasa (VKR) is one such classical herbo-mineral formulation listed in the ancient medicine text Bharat Bhaishajya Ratnakar [10] for its indication in diabetes. It consists of Suvarna Bhasma [11] (processed gold), Rajata Bhasma [12] (processed silver), Vanga Bhasma [13] (processed tin), Naga Bhasma [14] (processed lead), Kantaloha Bhasma [15] (processed iron), Abhraka Bhasma (processed mica) [16], Pravala Bhasma [17] (processed coral), Mouktik Bhasma [18] (processed pearl), Rasasindoor (sublimed red sulfide of mercury) [19] processed in cow milk, Ikshu (*Saccharum officinarum*) Swarasa, Vasa (*Adhatoda vasica*) Swarasa, Shweta (*Santalum album*) Kwath, Usheer (*Vetiveria zizanioides*) Kwath, Rheebera (*Pavonia odorata*) Kwath, Haridra (*Curcuma longa*) Kwath, Kadali (*Musa paradisiaca*) Kwath, Kamal Pushpa (*Nelumbium speciosum*) Swarasa, and Jati Pushpa (*Jasminum officinale*) Swarasa. VKR has shown beneficial effects in animal models of diabetes [20,21]. VKR treatment has also shown improvement in retinopathy in type 1 diabetic rats [22]. We have also studied the effect of VKR in animal models of type 2 diabetic cardiomyopathy [23].

There is a lack of comprehensive scientific investigation into the effectiveness of VKR in delaying or managing DE. Thus, the present study was designed to investigate the neuroprotective potential of VKR in high-fat diet (HFD) and streptozotocin (STZ)-induced type 2 DE in rat models. Type 2 diabetes was induced in rats by modification of diet for two weeks using HFD, followed by a single dose administration of streptozotocin (35 mg/kg, *i.p.*) which is an established animal model.

## Materials and methods

Chemicals and reagents

VKR tablets (batch no: P220500079) were received from Shree Dhootapapeshwar Ltd., India. Streptozotocin (STZ) (Lot No.: 022120029) was purchased from MP Biomedicals LLC (USA). The HFD (Batch No.: 20-41) composed of 60% fat, 20% carbohydrates, and 20% proteins was procured from VRK Nutritional Solutions, India. Diagnostic kits such as glucose, HbA1c, and C-reactive protein (CRP) were purchased from Transasia Biomedicals Ltd., India. Rat insulin (Cat #: KTE101059), TNF-α (Cat #: KET9007), IL-1β (Cat #: KTE9001), IL-6 (Cat #: KTE9004), and AchE (Cat # KTE100324) ELISA kits were procured from Abbkine Scientific Co., Ltd (USA). Dopamine hydrochloride and DL-norepinephrine hydrochloride were purchased from Sigma Aldrich (USA), and serotonin hydrochloride was procured from TCI Chemicals Pvt. Ltd., India. HPLC-grade methanol was procured from Advent Chembio Pvt. Ltd., India. Milli-Q water purification system (Elix® 3, Millipore, Darmstadt, Germany) was used to generate purified water required for the experimentation.

Experimental animals and study design

Male Sprague Dawley rats (180-200 g) were procured from the National Institute of Biosciences in Pune, Maharashtra, India. These rats were housed at a controlled temperature of 22 ± 2°C and a light/dark cycle of 12 hours and provided food and water *ad libitum*.

After a week of acclimatization, the rats were randomly divided into five groups. Group I (normal control) animals were fed with a normal diet and water ad libitum. Group II (disease control) animals were fed with HFD for two weeks; after that, a single dose of STZ (35 mg/kg, *i.p.*) was administered on the 15th day. Group III (diabetic control + glipizide 5 mg/kg) animals received HFD for two weeks, followed by a single dose of STZ (35 mg/kg, *i.p.*) on the 15th day, and then treated with glipizide (5 mg/kg, *p.o.*). Group IV (diabetic control + VKR 28 mg/kg) animals were fed with HFD for two weeks, and a single dose of STZ (35 mg/kg, *i.p.*) was administered on the 15th day of the study, followed by VKR (28 mg/kg, p.o.) treatment. Group V (diabetic control + VKR 56 mg/kg) animals were fed with HFD for two weeks, and a single dose of STZ (35 mg/kg, *i.p.*) was administered on the 15th day of the study, followed by VKR (56 mg/kg, *p.o.*) treatment. The doses of VKR were selected based on the dose used in clinical practice. Plasma glucose levels of animals were estimated after a week of STZ administration, and the animals with plasma glucose levels above 250 mg/dL were considered diabetic and were included further in the study. All the animals received HFD throughout the study period, except normal control animals. Animals in groups III, IV, and V received the treatment for 16 weeks [21]. At the end of the study, all the animals were humanely sacrificed using urethane (1.2 g/kg, *s.c.*).

Cognitive function test (Morris water maze test)

The Morris water maze (MWM) test was utilized to evaluate the spatial memory in experimental animals. A large, circular swimming apparatus, with a diameter of 150 cm and a height of 45 cm, was partitioned into four equal quadrants and filled with water to an appropriate level. During the acquisition phase, a submerged platform measuring 10 cm x 10 cm was positioned in the water, elevated 1 cm above the surface. The animals were introduced into one of the quadrants of the swimming tank and were permitted 120 seconds to locate the platform; assistance was provided if the subject failed to do so, after which the animal was allowed to remain on the platform for up to 30 seconds. Each animal underwent four trials daily over a span of four consecutive days as part of the training regimen. In the retention phase, the water was rendered opaque by incorporating milk powder to obscure the submerged platform, which was situated 1 cm below the water surface. Subsequently, each rodent was placed in the tank in a manner analogous to the acquisition phase, and the retention of memory was assessed. Escape latencies were recorded by measuring the duration taken by the animal to discover the concealed platform within the tank [22].

Estimation of biochemical parameters, AChE activity, serum Insulin, and homeostatic model assessment for insulin resistance (HOMA-IR)

Blood samples were collected in a microcentrifuge tube from the retro-orbital plexus of rats. The assessment of glycohemoglobin (HbA1c) was conducted on the whole blood samples as per instructions provided by Transasia Biomedicals Ltd., India. Serum insulin and AChE levels in the brain samples were analyzed using an ELISA kit (Abbkine, USA).

The homeostatic model assessment for insulin resistance (HOMA-IR) was calculated using the formula:

HOMA-IR = (Insulin X Glucose) / 22.5

The concentration of insulin was expressed in mIU/L and glucose in mmol/L.

Organ collection

Brain tissue was collected and stored at -80ºC and was used for the preparation of brain homogenate. Brain tissue was isolated for histopathological studies. The remaining portion of brain tissue was homogenized in 10 volumes of ice-cold 0.1 M phosphate buffer solution (pH 7.4) by using a probe homogenizer (Polytron PT 2500E, Kinematica, Switzerland). The tubes containing brain tissue were immersed in ice to avoid the rise in temperature. The total homogenate was centrifuged into two parts: the first part was centrifuged at 4,500 rpm (2,500 g) for 20 min at 4°C to prepare post-nuclear supernatant (PNS), and the other part was centrifuged at 9,000 rpm (10,000 g) for 20 min at 4°C to prepare post-mitochondrial supernatant (PMS) for biochemical assessment [23]

Determination of pro-inflammatory biomarkers

The CRP levels in the serum were estimated using a Transasia Biomedicals Ltd. (India) kit, and an estimation of TNF-α, IL-1β, and IL-6 was carried out in the brain homogenate using ELISA kits supplied by Abbkine, USA.

Determination of oxidative stress parameters

The protein content of the brain homogenate was measured according to Lowry’s method. The homogenate previously prepared was used to estimate the levels of malondialdehyde (MDA), superoxide dismutase (SOD), catalase (CAT), and reduced glutathione (GSH), as per the method described [21].

H&E staining

Brain tissues were stored in 10% neutral buffered formalin and were processed for histopathology analysis. Formalin-fixed tissues of the brain were trimmed longitudinally and routinely processed. Tissue processing was done to dehydrate in ascending grades of alcohol, clearing in xylene and embedding in paraffin wax, followed by fixation in the paraffin blocks. Thin transverse sections of 5 μm thickness of the hippocampus and cortex region were taken using a microtome. The sections were stained with H&E and examined under a digital microscope 400X (Motic, Canada) [21].

Estimation of neurotransmitters

An isocratic fluorescence-based high-performance liquid chromatography (HPLC) method was used for analysis. The analysis was carried out using the HPLC System, LC-2010 CHT (Shimadzu Corporation, Kyoto, Japan). LabSolution ® software (Shimadzu Corporation, Kyoto, Japan) was used for data recording and processing. Thermoscientific ® 4.6 mm x 50 mm C18 (250 x 4.6 mm, 5 μm) column was used to achieve chromatographic separation. The column temperature was maintained at 35°C, and the flow rate was set at 0.8 mL/min throughout the analysis. The mobile phase was acetate buffer (pH 3.5, 12 mM acetic acid, 0.26 mM Na2 EDTA)-methanol (90:10 v/v). Isolated tissues were homogenized in 200 µL diluent using probe sonication (three cycles, 25 seconds each, 40% amplitude), followed by centrifugation at 8,000 RPM for 15 minutes. Later, the 10 µL supernatant sample was injected into the HPLC system. The fluorescence was monitored at excitation and emission wavelengths of 279 nm and 320 nm, respectively. NE, 5-HT, and DOPA were identified by drawing their comparison between retention time in the sample (tissue extracts) with that of the standard solution. The calibration curve of the standard DA, 5-HT, and NE (0.2, 0.4, 0.6, 0.8, 1.0, 1.2 µg/mL) was found to be linear with regression coefficients (R2) of 0.9983, 0.9989, and 0.992, respectively.

Statistical analysis

All the data were expressed as mean ± SEM. Statistical analysis was carried out using GraphPad Prism 8 software (GraphPad Software, San Diego, CA). One-way ANOVA followed by Dunnett's multiple comparison test was used for analysis. p<0.05 was kept as the level of significance.

## Results

Effect of VKR on escape latency

**Figure 1:**
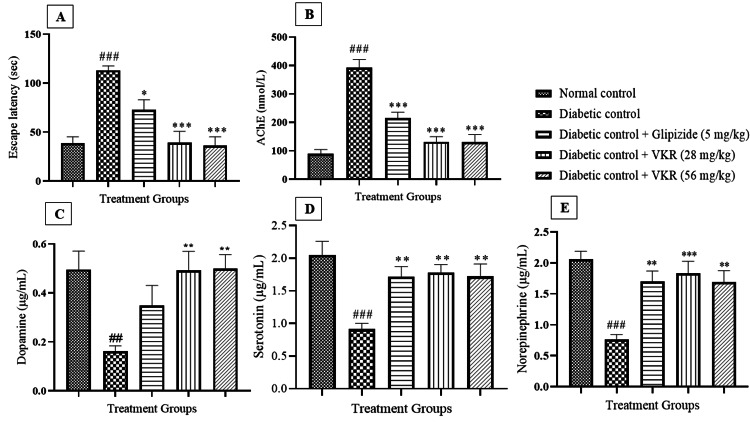
Effect of VKR on escape latency, AChE, dopamine, serotonin, and norepinephrine levels. All values are expressed as mean ± S.E.M. (n=06). ###p<0.001, ##p<0.01 when the diabetic control group was compared with the normal control group. ***p<0.001, **p<0.01, and *p<0.05 when the treatment groups were compared with the diabetic control group.

Animals in the diabetic control group took more time to reach the platform and hence exhibited a significant (p<0.001) increase in escape latency time as compared to normal control animals. Animals in the glipizide (5 mg/kg) and VKR (28 mg/kg and 56 mg/kg) treated group exhibited a significant (p<0.05, p<0.001) decrease in the escape latency time when compared with the animals in the diabetic control group (Figure 1A).

Effect of VKR on plasma glucose, glycated hemoglobin, serum insulin, and HOMA-IR

The plasma glucose levels, glycated hemoglobin, serum insulin, and HOM-IR levels were significantly (p<0.001) higher in the diabetic control animals when compared with the animals in the normal control group. On the other hand, animals in all the treated groups showed a significant (p<0.001) decrease in plasma glucose levels, glycated hemoglobin, serum insulin, and HOM-IR levels when compared with the animals in the diabetic group (Table 1).

**Table 1:** Effect of VKR on glycaemic profile, pro-inflammatory markers, and oxidative stress markers. All values are expressed as mean ± S.E.M. (n=6). ###p<0.001, ##p<0.01 when the diabetic control group was compared with the normal control group. ***p<0.001, **p<0.01, and *p<0.05 when the treatment groups were compared with the diabetic control group.

Groups	Normal Control	Diabetic Control	Diabetic + Glipizide (5 mg/kg)	Diabetic + VKR (28 mg/kg)	Diabetic + VKR (56 mg/kg)
Plasma glucose (mg/dL)	103.7 ± 5.73	461.7 ± 13.03^###^	148.3 ± 4.36***	236.7 ± 17.08***	221.8 ± 17.50***
HbA1c (%)	4.830 ± 0.11	12.33 ± 0.50^###^	6.19 ± 0.46***	7.58 ± 0.85***	6.41 ± 0.40***
Serum insulin (mU/L)	1.02 ± 0.19	4.13 ± 0.33	1.50 ± 0.08^###^	1.82 ± 0.10***	1.511 ± 0.09***
HOMA - IR	0.188 ± 0.03	4.39 ± 0.51^###^	0.55 ± 0.04***	1.09 ± 0.09***	0.73 ± 0.05***
Serum C-RP (mg/dL)	0.84 ± 0.03^###^	1.58 ± 0.04^###^	1.30 ± 0.07*	1.26 ± 0.08**	1.20 ± 0.04***
TNF-α (pg/ml)	49.24 ± 5.24	202.0 ± 27.86^###^	99.26 ± 10.08**	94.91 ± 21.39***	89.58 ± 12.04***
IL-6 (pg/ml)	253.0 ± 21.98	588.8 ± 27.19^###^	326.3 ± 33.28***	293.0 ± 15.64***	272.2 ± 17.78***
IL-1β (pg/ml)	568.2 ± 61.61	1433 ± 130.7^###^	1020 ± 55.40*	1009 ± 133.9*	950.7 ± 79.27*
MDA (μMol/mg of protein)	9.64 ± 1.51	48.16 ± 9.53^###^	27.27 ± 6.43*	17.75 ± 4.09**	15.46 ± 2.97**
CAT (μm of H_2_O_2 _decomposed/min/mg of protein)	60.17 ± 7.59	18.61 ± 2.67^###^	40.40 ± 4.05	48.49 ± 4.05**	53.88 ± 5.19***
SOD (U/mg of protein)	4.58 ± 0.65	0.88 ± 0.08^###^	2.62 ± 0.37	2.81 ± 0.34*	3.83 ± 0.49***
GSH (μMol/mg of protein)	156.8 ± 28.49	47.61 ± 6.52^##^	102.1 ± 11.94	126.7 ± 19.51*	118.1 ± 19.92*

Effect of VKR on brain AChE activity and pro-inflammatory markers

The levels of AChE, serum CRP, TNF-α, IL-1β, and IL-6 were significantly (p<0.001) increased in diabetic control animals as compared to the normal control group. VKR at both the doses and glipizide (5 mg/kg) significantly (p<0.001) reduced the AChE activity and IL-6 levels when compared with the animals in the diabetic group (Figure 1B). VKR treatment at 28 mg/kg showed a significant (p<0.01, p<0.001, p<0.05) reduction in CRP, TNF-α, and IL-1β levels when compared to the diabetic group. Additionally, VKR (56 mg/kg) treated animals exhibited significant (p<0.001, p<0.05) decrease in CRP, TNF-α, and IL-1β levels respectively as compared to diabetic animals. Glipizide (5 mg/kg) treated group also a significant (p<0.05, p<0.01, p<0.05) reduction in CRP, TNF-α, and IL-1β levels when compared with the diabetic control animals (Table 1).

Effect of VKR on brain oxidative stress markers

The MDA level was found to be significantly (p<0.001) high, while CAT, SOD, and GSH showed a significant (p<0.001, p<0.01) decrease in the diabetic control group as compared to normal control animals. Treatment with VKR (28 mg/kg and 56 mg/kg) significantly (p<0.01) reduced the increased MDA levels. Additionally, CAT, SOD, and GSH levels were significantly (p<0.05, p<0.01, p<0.001) increased in both the VKR-treated group as compared to animals in the diabetic control group (Table 1). Glipizide (5 mg/kg) did not show any significant decrease in CAT, SOD, and GSH levels as compared to the animals in the diabetic control group.

Effect of VKR on histopathology

**Figure 2:**
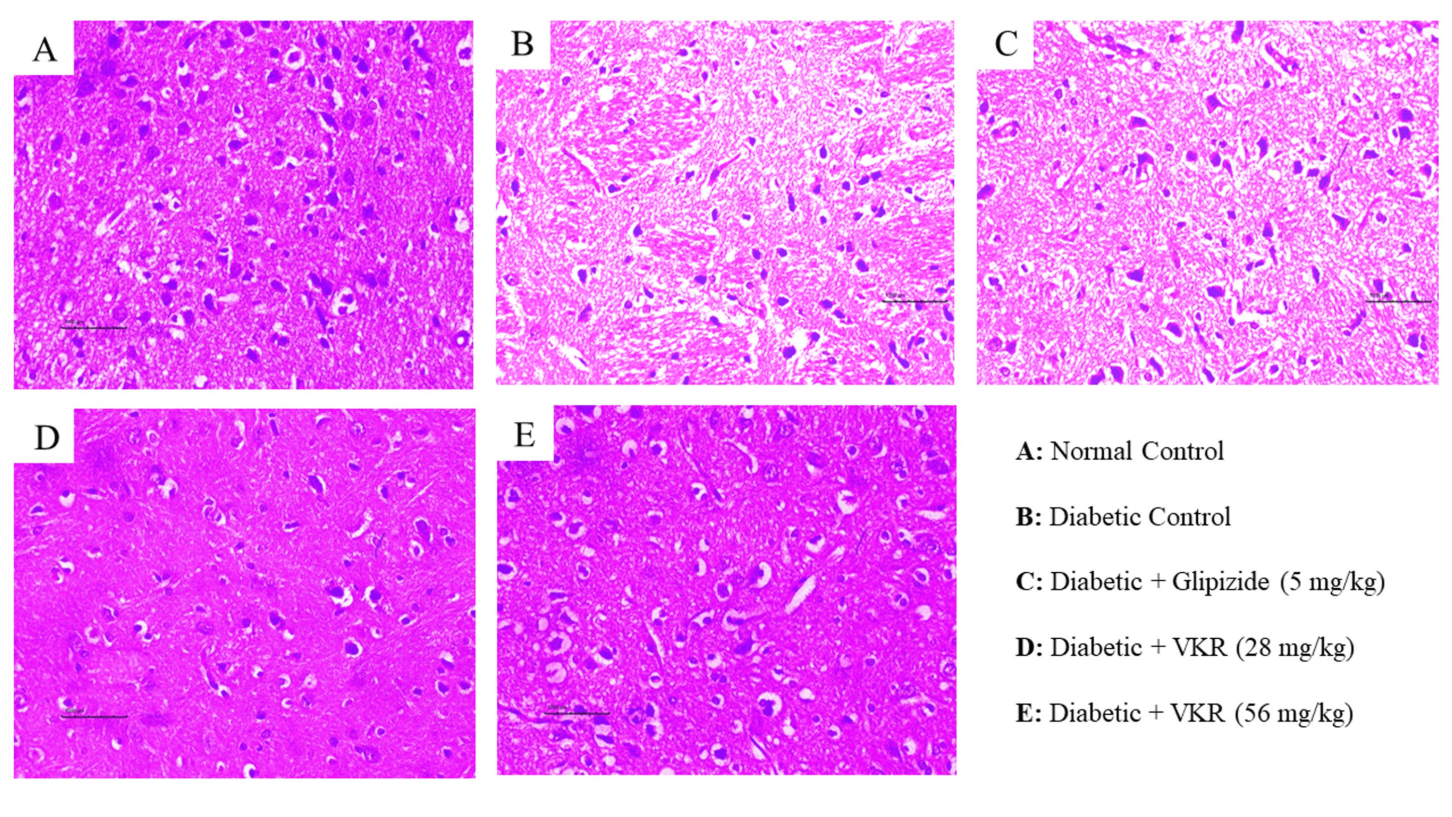
Effect of VKR on brain tissue histopathology (H&E, 400X). A: Normal control, normal neuronal cells. B: Diabetic control showing moderate neuronal degeneration with pyknotic nuclei, a reduced layer of neuronal cell. C: Glipizide (5 mg/kg) treated animals showing mild neuronal cells with pyknotic nuclei. D: VKR (28 mg/kg) showing mild neuronal degeneration with pyknotic nuclei, a reduced layer of neuronal cell. E: VKR (56 mg/kg) showing minimal neuronal degeneration, a reduced layer of neuronal cell.

The diabetic control group showed marked histopathological changes such as multifocal moderate neuronal degeneration with pyknotic nuclei and multifocal moderate reduced layer of neuronal cells in the brain when compared with the normal control group. Treatment with VKR at both dose levels showed a reduction in neuronal degeneration and displayed lesser histopathological damage within the layer of neuronal cells when compared with the diabetic control group (Figure 2).

Effect of VKR on the neurotransmitter level

The levels of DA, 5-HT, and NE were significantly (p<0.01, p<0.001) low in the diabetic control group as compared to animals in the normal control group. The glipizide 5 mg/kg group showed a significant (p<0.01) increase only in 5-HT and NE levels when compared to the diabetic group. VKR treatment showed a significant (p<0.01) increase in the DA and 5-HT levels as compared to diabetic animals at both dose levels. VKR (28 mg/kg, 56 mg/kg) showed a significant (p<0.001, p<0.01, respectively) increase in the NE level when compared to the diabetic group (Figures 1C, 1E, 1D).

## Discussion

The objective of the present study was to evaluate the role of VKR in cognitive impairment associated with type 2 diabetes in rats. The presently used HFD/STZ animal model exhibited an increase in cognitive decline, a surge in inflammatory biomarkers, alteration in neurochemical changes, antioxidant enzyme markers and neurotransmitter levels, and changes in histopathological characteristics [24]. Glucose is the key source of energy that pumps the healthy brain’s metabolic and synaptic plasticity; on the contrary, excess glucose can be damaging. VKR administration at both doses considerably reduced the glycaemic indices. There is a considerable amount of compelling evidence indicating that hyperglycemia is a causative factor for cognitive impairment [25].

In the present study, escape latency was recorded as a parameter to assess spatial cognition using the Morris water maze test. The results from this test indicated that a decline in cognitive behavior was more prevalent in the diabetic control group, and the administration of VKR at both doses over a period of 16 weeks markedly reduced the time taken to escape; a similar effect was found in the glipizide 5 mg/kg treated rats. This observed decrease in escape latency can be interpreted as a positive outcome, suggesting an improvement in spatial memory, and learning abilities due to the mitigated effects of hyperglycemia. The brain expresses a significant amount of cholinergic neuro-activities that have an influence on the process of learning and memory by modulating neuronal excitability, synaptic transmission, and plasticity [26]. The synthesis of acetylcholine and a decrease in AChE activity is essential for normal cognitive functioning. However, an elevation in AChE activity may potentially compromise the cognitive capabilities of neurons [27,28]. In this study, an escalation in AChE activity was observed in the untreated diabetic group, indicating a decline in cholinergic function due to the depletion of acetylcholine activity in the brains of diabetic rats. Remarkably, animals in the VKR-treated (28 mg/kg and 56 mg/kg) group demonstrated lower levels of AChE activity.

Diabetes exhibited elevated levels of pro-inflammatory cytokines within the cerebral regions, consequently leading to neuronal damage. A recent study showed that spatial-recognition memory deficits were observed in db/db mice afflicted with diabetes and obesity, which were correlated with increased pro-inflammatory cytokine levels, namely, IL-1β, TNF-α, and IL-6, suggesting a connection between inflammation and memory deterioration [29]. In the presence of inflammation, immunogenic molecules can incite microglia activation, resulting in the augmentation of cytokine and reactive oxygen species (ROS) generation, leading to the progression of cognitive impairment [30]. In the present study, the HFD/STZ administration in rats resulted in a significant increase in the pro-inflammatory cytokines TNF-α, IL-6, and IL-1β levels within the brain of the diabetic control group. Treatment with VKR at both doses demonstrated a substantial reduction in brain TNF-α, IL-6, and IL-1β levels when compared to the diabetic control group.

The progression of diabetes has been correlated with the presence of oxidative stress and the occurrence of hyperglycemia triggers the formation of ROS. Elevated levels of oxidative stress have been associated with the emergence of cognitive impairments in diabetic rats [31]. It is widely acknowledged that hyperglycemia-induced neurotoxicity primarily arises from high production of advanced glycation end (AGE) products, increased flux in the polyol pathway, activation of protein kinase C (PKC) isoforms, and augmented flux in the hexosamine pathway, all of which contribute to an augmentation in oxidative harm and vascular complications [32]. DE includes neuronal dysfunction resulting from glucose toxicity, a pathological state characterized by persistent hyperglycemia that significantly induces oxidative stress and neuroinflammatory responses, ultimately resulting in neuronal death [33]. In this study, the induction of diabetes resulted in a notable increase in MDA concentrations and a decrease in GSH, SOD, and CAT activity in the brain of untreated diabetic rats when compared to the control group. However, this finding was counteracted by the administration of VKR at both doses, as demonstrated by the significant decrease in brain MDA levels and the increase in GSH, SOD, and CAT activity in comparison to the diabetic control group. The glipizide 5 mg/kg group showed a non-significant impact on the oxidative stress parameters. In the disease control group, examination of brain tissue stained with hematoxylin and eosin displayed a marked presence of neurodegeneration characterized by pyknotic nuclei. The administration of VKR for 16 weeks proved effective in ameliorating these degenerative alterations in the regions of the brain owing to its neuroprotective attributes.

The interconnection between the brain's monoamine neurotransmitter system and glucose regulation has been extensively explored in scientific literature. Both type 1 diabetes mellitus (T1DM) and type 2 diabetes mellitus (T2DM) are associated with detrimental changes in the brain's monoaminergic system, which contribute to diabetes-related neurodegeneration [33]. Numerous research groups have reported alterations in the metabolism and levels of NE, 5-HT, and DA in specific regions of the brain in diabetic rodents. A murine model of T2DM induced by a prolonged HFD exhibited a decrease in serotonin levels in the hippocampus and an overactivation of inhibitory serotonin autoreceptors in the dorsal raphe nuclei, resulting in a disruption of the serotonergic circuit [34]. These detrimental changes contribute to mood disorders and eating disorders associated with T2DM. Additionally, experimental evidence suggests a relationship between glucose metabolism and the dopaminergic system. Studies have shown that modulation of DA levels in the striatum and throughout the body influences overall glucose metabolism and energy balance in rodents [35]. Conversely, the presence of diabetes promotes neurodegeneration and impairs dopaminergic neurotransmission. This aligns with the understanding that conditions such as diabetes, hyperglycemia, and relative insulin deficiency can impact the dopaminergic system functioning [23]. Dysregulation of insulin signaling within the central nervous system modifies the functionality of both neuronal and glial cells at the synaptic interface and is correlated with neurodegenerative diseases and cognitive impairments [36] [37]. In fact, insulin serves as a crucial regulator of neuronal survival and DA metabolism [38,39]. In both doses of the VKR-treated group, animals exhibited a significant rise in brain monoamine levels, specifically DA, 5-HT, and NE in comparison to the diabetic control group. Similar findings were observed in the glipizide-treated group, except for DA.

## Conclusions

The effect of VKR in HFD and STZ-induced diabetic encephalopathy was evaluated by various experimental studies performed in the present work. Nevertheless, it is imperative to conduct more detailed investigations into the specific mechanisms underlying genetic expression to further substantiate the understanding of the therapeutic effects that VKR may have on the pathogenesis of diabetic encephalopathy, as this will provide a more comprehensive insight into its potential benefits. From the findings derived from this study, it can be concluded that prolonged administration of VKR holds significant therapeutic potential in the management of diabetic encephalopathy.
